# Agonist-induced internalization and desensitization of the apelin receptor

**DOI:** 10.1016/j.mce.2016.07.040

**Published:** 2016-12-05

**Authors:** George R. Pope, Sharada Tilve, Craig A. McArdle, Stephen J. Lolait, Anne-Marie O'Carroll

**Affiliations:** Laboratories for Integrative Neuroscience and Endocrinology, School of Clinical Sciences, University of Bristol, Whitson Street, Bristol BS1 3NY, UK

**Keywords:** G protein-coupled receptor, Apelin, Apelin receptor, Intracellular trafficking, Signalling, Extracellular-signal-regulated kinase (ERK), APJ, apelin receptor, βARR, β-arrestin1, BIM, bisindolylmaleimide I, CME, clathrin-mediated endocytosis, CCV, clathrin coated vesicle, DNM, dominant-negative mutant, DYN, dynamin, eGFP, enhanced green fluorescent protein, FCS, fetal calf serum, GRK, G protein-coupled receptor kinase 2, m, mouse, NGS, normal goat serum, P, penicillin, P70S6K, S6 ribosomal protein kinase, PCSE, proportional cell surface expression, PTX, pertussis toxin, S, streptomycin, U0126, 1,4-diamino-2,3-dicyano-1,4-bis[2-aminophenylthio] butadiene

## Abstract

Apelin acts via the G protein-coupled apelin receptor (APJ) to mediate effects on cardiovascular and fluid homeostasis. G protein-coupled receptor (GPCR) trafficking has an important role in the regulation of receptor signalling pathways and cellular functions, however in the case of APJ the mechanisms and proteins involved in apelin-induced trafficking are not well understood. We generated a stable HEK-293 cell line expressing N-terminus HA-tagged mouse (m) APJ, and used a semi-automated imaging protocol to quantitate APJ trafficking and ERK1/2 activation following stimulation with [Pyr^1^]apelin-13. The mechanisms of [Pyr^1^]apelin-13-induced internalization and desensitization were explored using dominant-negative mutant (DNM) cDNA constructs of G protein-coupled receptor kinase 2 (GRK2), β-arrestin1, EPS15 and dynamin. The di-phosphorylated ERK1/2 (ppERK1/2) response to [Pyr^1^]apelin-13 desensitized during sustained stimulation, due to upstream APJ-specific adaptive changes. Furthermore, [Pyr^1^]apelin-13 stimulation caused internalization of mAPJ via clathrin coated vesicles (CCVs) and also caused a rapid reduction in cell surface and whole cell HA-mAPJ. Our data suggest that upon continuous agonist exposure GRK2-mediated phosphorylation targets APJ to CCVs that are internalized from the cell surface in a β-arrestin1-independent, EPS15- and dynamin-dependent manner. Internalization does not appear to contribute to the desensitization of APJ-mediated ppERK1/2 activation in these cells.

## Highlights

• Agonist exposure induces internalization and reduction in cell surface APJ levels.
• ERK1/2 response to [Pyr^1^]apelin-13 desensitizes rapidly during sustained stimulation.
• Desensitization of ERK1/2 response is due to upstream APJ-specific adaptive changes.
• Internalization is EPS15-, dynamin- and GRK2-dependent but β-arrestin-independent.
• Desensitization response is independent of dynamin, GRK2 and β-arrestin.

## 1 Introduction

The apelin gene encodes a preproprotein of 77 amino acids that is processed into multiple shorter peptides including apelin-36, apelin-17, apelin-13 and apelin-12 (Tatemoto et al., 1998). Apelin-13 may undergo post-translational modification leading to the formation of a more stable and biologically active pyroglutamyl form, [Pyr^1^]apelin-13. Apelin acts via the single apelin receptor (APJ) subtype to mediate effects on the cardiovascular system (Reaux et al., 2001, Ishida et al., 2004), fluid homeostasis (O'Carroll and Lolait, 2003), glucose metabolism (Dray et al., 2008), and food intake (Taheri et al., 2002), influencing not only cAMP production but also PKC, PI3K, protein kinase B (Akt), S6 ribosomal protein kinase (p70S6K), ERK (Masri et al., 2002, Masri et al., 2004) and cytoplasmic Ca^2+^ concentration (Choe et al., 2000). APJ couples to G_i/o_ in assays measuring extracellular acidification rates (Hosoya et al., 2000) and phosphorylation of ERK and p70S6 kinase (Masri et al., 2002, Masri et al., 2004), and activates ERK1/2 and inhibits adenylate cyclase through Gα_i1-_ and Gα_i2_-dependent pathways (Masri et al., 2006, Bai et al., 2008). However apelin activation of ERK1/2 is mediated via PKC in HEK293 cells expressing mouse APJ, indicative of coupling to either G_o_ or G_q/11_ (Masri et al., 2002). Additionally the beneficial inotropic effect of apelin *in vivo* is only partially abrogated by pertussis toxin (PTX) and by PKC inhibitors, indicating that some of the actions of APJ could be mediated by G_i/o_ and/or G_q/11_ coupling (Szokodi et al., 2002). Recently it has been shown that mechanical stretch signals via APJ to induce myocardial hypertrophy by a G protein-independent, β-arrestin-dependent pathway (Scimia et al., 2012). Interestingly APJ, when stably expressed in CHO cells, shows ligand bias with endogenous ligands as, for example, apelin-13 preferentially signals to ERK via Gα_i2_ whereas apelin-36 does so equally well via Gα_i1_ and Gα_i2_ (Masri et al., 2006).

As with most G protein-coupled receptors (GPCRs), sustained activation of APJ can cause desensitization and this has been reported to occur for APJ-mediated effects on cytoplasmic Ca^2+^ concentration, as well as for effects on activity of adenylyl cyclase, ERK and Akt (Ishida et al., 2004, Masri et al., 2006). APJ also undergoes agonist-induced internalization and down-regulation and so research has focused on the possible role for the canonical pathway for rapid homologous receptor desensitization and trafficking in mediating adaptive responses to APJ activation (Evans et al., 2001, Zhou et al., 2003, Lee et al., 2010). In this pathway, agonist occupied GPCRs are preferred substrates for phosphorylation by G-protein receptor kinases (GRKs) and this phosphorylation mediates binding with β-arrestins that prevent the receptors from activating their cognate G-proteins, thereby causing receptor desensitization. The β-arrestins also target the desensitized receptors for internalization via clathrin-coated vesicles (CCVs). After this the vesicles are uncoated, β-arrestins dissociate, receptors are dephosphorylated and the receptor-containing vesicles may be trafficked back to the plasma membrane (a process that can mediate resensitization to the agonist) or to lysosomes for proteolytic digestion (a process that can cause receptor down-regulation). Differing patterns of β-arrestin interaction have allowed the sorting of GPCRs into two classes: Class A receptors, that have a brief interaction with β-arrestins (at the plasma membrane) and preferentially bind β-arrestin2 over -1, and display rapid recycling; and Class B receptors, that form a stable complex with both β-arrestins with equal affinity, and which internalize with the β-arrestins into endosomes. Additional players in this process include epsin and EPS15, which act as adapter proteins for clathrin-mediated endocytosis (CME) (Wolfe and Trejo, 2007), and dynamin, a GTPase that forms a multimeric complex around the neck of nascent endocytic vesicles and mediates their budding off to form endosomes (Damke, 1996).

The adaptive processes outlined above are thought to be relevant for APJ as apelin causes clathrin-mediated APJ internalization (Reaux et al., 2001, El Messari et al., 2004) and also translocation of β-arrestin1 and -2 to the cell surface, indicating translocation to phosphorylated APJ (Lee et al., 2010). Moreover, after agonist-induced internalization, APJ can either be recycled to the cell surface or be degraded in lysosomes (Lee et al., 2010). Interestingly, APJ trafficking displays ligand bias for both Class A and B β-arrestin/recycling behaviour as when internalization is stimulated by [Pyr^1^]apelin-13, internalized APJ is rapidly recycled to the plasma membrane with none remaining in the cytoplasm at 60 min, whereas APJ is retained within the cell for up to 120 min after apelin-36-stimulated internalization (Zhou et al., 2003). Similarly, although apelin-13 causes β-arrestin1 translocation to the plasma membrane, the internalized receptors are not associated with β-arrestin1 and are rapidly recycled to the cell surface via early endosomes (Evans et al., 2001, Lee et al., 2010), whereas after apelin-36 stimulation the internalized APJ are co-localized with β-arrestin1 and then undergo rab-7-dependent trafficking to lysosomes (Lee et al., 2010). Finally, truncation of the APJ C-terminus (in order to delete potential GRK phosphorylation sites) prevents homologous desensitization to effects of apelin-13, but not to those of apelin-36, on inhibition of adenylyl cyclase and activation of ERK and Akt (Masri et al., 2006, Lee et al., 2010).

Apelin/APJ has emerged as a major signalling pathway in physiological homeostasis (O'Carroll et al., 2013) and central to ascertaining the precise function of this receptor is an understanding of the system of regulation that dynamically modulates APJ signalling. In peripheral tissues the apelinergic system appears to be down-regulated in hypertensive disease – levels of apelin immunoreactivity in plasma, and in ventricular and aortic tissues, are lower in the spontaneously hypertensive rat, a genetic model of hypertension, than in control Wistar-Kyoto normotensive rats (Zhang et al., 2006b, Zhang et al., 2006a, Zhong et al., 2005). Additionally circulating levels of apelin are decreased in patients with essential (Sonmez et al., 2010) and pulmonary (Chandra et al., 2011) hypertension, while there is a negative correlation between plasma apelin levels and blood pressure (Zhu et al., 2013). This suggests a role for decreased peripheral apelin signalling in the pathophysiology of hypertension. Receptor trafficking is a key process for regulating receptor signalling pathways and cellular functions, however in the case of APJ the mechanisms and proteins involved in agonist-induced trafficking are not well understood. To further understand the signalling and regulation of APJ, and thus the efficacy of ligands for potential therapeutic intervention, this study set out to characterize the mechanisms underlying [Pyr^1^]apelin-13-induced APJ desensitization and internalization, and to determine whether agonist-induced APJ internalization contributes to its functional desensitization. A stable HEK-293 cell line expressing N-terminus HA-tagged mouse APJ (mAPJ) was generated, and a semi-automated imaging protocol was used to quantitate ERK1/2 activation and APJ trafficking in this cell line following agonist activation with [Pyr^1^]apelin-13. The mechanisms of [Pyr^1^]apelin-13-induced internalization were further explored using dominant-negative mutant (DNM) cDNA constructs of GRK2 (GRK^DNM^), β-arrestin1 (βARR^DNM^), EPS15 (EPS^DNM^) and dynamin (DYN^DNM^), known effectors of CME.

## 2 Methods and materials

### 2.1 Materials and cell culture

DMEM, FCS, penicillin (P), streptomycin (S), normal goat serum (NGS), Alexa Fluor 488 goat anti-mouse IgG (H + L) and Alexa Fluor 546 goat anti-rabbit IgG (H + L) were purchased from Life Technologies (Paisley, UK). cDNA encoding DNMs (provided by Professor Eamonn Kelly, University of Bristol) included GRK^DNM^ (K220R), βARR^DNM^ (319–418), EPS^DNM^ (EΔ95/295) and DYN^DNM^ (K44A). Anti-HA antibody was from Cambridge Bioscience (Cambridge, UK); rabbit anti-ERL1/2 antibody was from Cell Signalling Technology UK; Hercules II Fusion DNA polymerase was from Agilent Technologies (Stockport, UK), Nanofectamin was purchased from PAA Laboratories (Somerset, UK), and 4′,6-diamidino-2-phenyindole (DAPI), adrenaline, EGF and mouse anti-ppERK1/2 antibody were from Sigma-Aldrich (Dorset, UK). [Pyr^1^]apelin-13 was purchased from Bachem (Bubendorf, Switzerland). Pertussis toxin (PTX), bisindolylmaleimide I (BIM) and 1,4-diamino-2,3-dicyano-1,4-bis[2-aminophenylthio] butadiene (U0126) were from Merck Chemicals (Nottingham, UK).

HEK293 cells, unless otherwise stated, were cultured in 10% FCS-supplemented DMEM containing glutamine (4 mM) and P/S (500 units/ml; 0.5 mg/ml). Cultures were maintained at 37 °C in 5% CO_2_. For imaging studies cells were seeded at 17,500 per well into Costar black-walled 96-well plates (Corning, Arlington, UK).

### 2.2 Stable and transient transfection

Untagged and HA-tagged mouse (m)APJ cDNAs were generated by PCR using 150 ng mouse 129SV genomic DNA (PCR conditions: 95 °C 2 min; 40 cycles of: 94 °C 45 s, 50 °C 1 min, 72 °C 1 min; and final extension of 72 °C 10 min) using Hercules II Fusion DNA polymerase. The integrity of the cDNA constructs was verified by DNA sequencing. Primers for the untagged receptor were directed to 5′ and 3′-regions of mAPJ and corresponded to 8462–10,285 bp of the mouse APJ gene (Genbank Accession number AC117228.2), generating a 1824 bp product. Primers for the tagged receptor were also directed to 5′ and 3′ regions of the receptor, but the 5′ primer contained an additional 27 bp, which coded for the Influenza HA epitope tag and generated a 1851 bp mouse product. The mAPJ gene, in the pcDNA3.1(+) vector (containing the neomycin resistance gene), was transfected into HEK293 cells by a calcium phosphate procedure (Chen and Okayama, 1988) and selected by G418. Stable cell lines highly expressing APJ were selected by Northern dot blot hybridization. Transient transfection of DNM cDNAs (0.4 μg/well) was performed with Nanofectamin according to the manufacturer's protocol, with DNM cDNA-containing medium removed after 4 h and replaced with fresh DMEM (0.1% FCS). Alongside each transfection, transfection efficiency was estimated using a 5-bromo-4-chloro-3-indolyl-β-D-galactopyranoside (X-gal) staining assay. Control cells were transfected with a mammalian vector inserted with a LacZ gene (pSV-β-Galactosidase control vector; Promega, UK), and subsequent beta-galactosidase (β-gal) activity estimated from the percentage of blue cells. Approximately 40% transfection efficiency was observed with Nanofectamin with HEK293 cells, that did not deviate significantly between experiments.

### 2.3 Receptor imaging studies

The APJ of HA-mAPJ-HEK293 cells have exofacial HA tags enabling cell surface receptor expression to be quantified with anti-HA antibody added to non-permeabilized cells. Cells were incubated in the presence or absence of [Pyr^1^]apelin-13 in DMEM (0.1% FCS), washed with ice-cold PBS and incubated with mouse anti-HA primary antibody (1:1000 dilution; 1 h). Whole cell APJ levels were also measured by immunohistochemistry but in this case the cells were permeabilized before addition of the primary antibody.

For quantification of HA-mAPJ recovery, HA-mAPJ-HEK293 cells were incubated in the presence or absence of [Pyr^1^]apelin-13 for 2 h, washed, then incubated in fresh medium as indicated in figure legends, before determination of either cell surface or whole cell APJ levels using anti-HA antibody. This 2 h time point is consistent with that used previously to promote APJ internalization (Masri et al., 2006).

APJ internalization was measured by labelling cell surface HA-mAPJ with primary antibody and then washing to remove unbound anti-HA antibody before stimulation with agonist. For one series of experiments clathrin-mediated internalization was blocked with hypertonic sucrose. In this case cells were incubated in physiological salt solution (NaCl (127 nM), NaH_2_PO_4_H_2_O (0.5 mM), CaCl_2_2H_2_O (1.8 mM), MgCl_2_ (2 mM), KCl_2_ (5 mM), NaHCO_3_ (5 mM), HEPES (10 mM), BSA (0.1%) glucose (10 mM), pH 7.4) with or without 0.4 M sucrose for 30 min prior to agonist stimulation, and washing in ice-cold PBS.

To measure recycling of internalized APJ to the cell surface, HA-mAPJ-HEK293 cells were incubated with primary antibody, washed with PBS and incubated in the presence or absence of [Pyr^1^]apelin-13 for 2 h. After aspiration of the agonist containing medium and two washes with PBS, cells were incubated with fresh medium as indicated in the figure legends.

For all the above experimental treatments, cells were subsequently fixed (2% paraformaldehyde/PBS, 30 min), permeabilized (pre-chilled methanol at −20 °C, 5 min) and washed (3×) with PBS. Following washing cells were blocked (5% NGS in PBS, 2 h), and incubated with secondary antibody (Alexa Fluor 488-conjugated goat anti-mouse IgG at 1:500 dilution in PBS with 1% NGS, 90 min). Cells were then washed (3×) with PBS, incubated with 300 nM DAPI for 15 min, and washed (2×) in PBS.

### 2.4 ERK phosphorylation assay

Cell expression of total (tERK) and di-phosphorylated ERK (ppERK) was visualized in stably transfected HEK293 cells with an immunocytochemistry protocol employing anti-tERK and -ppERK antibodies. Quantification of ERK phosphorylation was performed by incubating mAPJ-HEK293 cells at 37 °C with [Pyr^1^]apelin-13 (100 nM) in DMEM (0.1% FCS) for 5 min. To explore homologous and heterologous desensitization mAPJ-HEK293 cells were preincubated for 2 h with medium in the presence or absence of [Pyr^1^]apelin-13. This time point has been used in previous studies on desensitization of APJ (Masri et al., 2006). Cells were then washed (×2) with PBS and exposed either to a second application of [Pyr^1^]apelin-13 (100 nM, 5 min) or to other ERK inducers, (adrenaline (1 μM), EGF (100 ng/ml), 5 min). Resensitization was monitored by varying the period between primary and secondary agonist incubation. For assays with DNM cDNAs, mAPJ-HEK293 cells were transiently transfected with DNM cDNAs before incubation with [Pyr^1^]apelin-13.

After experimental treatment cells were immunostained with primary antibody (mouse anti-ppERK1/2 (1:1600 dilution) or rabbit anti-ERK1/2 (1:800 dilution) in 1% NGS in PBS, 4 °C, overnight). Cells were then washed (3×) with PBS, and incubated with secondary antibody (Alexa Fluor 488 goat anti-mouse IgG (H + L) or Alexa Fluor 546 goat anti-rabbit IgG (H + L) (1:500 dilution, 90 min)). Cells were washed (3×) with PBS, stained with DAPI, and washed (2×) with PBS as above, before imaging. Responses for desensitization experiments are expressed as a percentage of maximal response, where the maximal response is defined as cells pre-treated with vehicle and then stimulated with [Pyr^1^]apelin-13.

### 2.5 Semi-automated image acquisition and analysis

Assays were quantified by semi-automated acquisition of digital fluorescence images using a high content imaging platform (IN Cell Analyzer 1000, GE Healthcare UK) and validated algorithms for image segmentation and quantification (IN Cell Analyzer version 1.0 software) as described (Finch et al., 2008). Digital images were taken with a 10× objective (Plan Apochromat, numerical aperture 0.45), with excitation and emission filters for each channel as follows, blue (360 ± 40 nm; 460 ± 40 nm), green (475 ± 20 nm; 535 ± 50 nm), and red (535 ± 50 nm; 620 ± 60 nm) using a 61002 trichroic mirror. Four fields were acquired per well (each field capturing a 0.602 mm^2^ area with a 10× objective), obtaining on average of 1000 cells per well.

For most experiments (cell surface or whole cell HA-mAPJ measures, and whole cell ppERK measures) image analysis software (In Cell 1000 Multi-target Analysis) was used to define the perimeter of the nucleus (from the DAPI stain) and the perimeter of the cell (from the HA or ppERK stain). Average fluorescence intensity over the entire cell area was calculated for each cell and background values (obtained with no primary antibody) were also determined. The figures show background subtracted and population averaged data in arbitrary fluorescence units (AFU). In most cases these are expressed as percentage of a vehicle control and for some experiments proportional cell surface expression was also calculated (PCSE; (cell surface expression ÷ whole cell expression) × 100). In receptor internalization assays, agonist exposure caused the internalized receptors to redistribute into punctate regions (presumably endosomes) in the cytoplasm and these “inclusions” were quantified using a Dual Area Analysis Algorithm (In Cell Analyzer version 1.0). The nuclear perimeter was determined from the DAPI stain and this was expanded with a 2 μm collar. The image analysis gave the number of inclusion over the collar and nucleus for each cell and figures show population averaged inclusion counts. The agonist-induced appearance of the antibody in puncta is consistent with the wealth of data showing agonist-induced internalization of these and other GPCRs. We have previously used this methodology to investigate agonist-induced internalization of gonadotropin-releasing hormone receptors (Finch et al., 2009).

### 2.6 Statistical analysis

IN Cell Analyser 1000 experiments were performed in 3 replicate wells with triplicate fields within each well, and experiments were performed at least 3 times. Data are expressed in figures as mean ± SEM. Statistical analysis was with a one-way ANOVA and *post hoc* Dunnett's test with GraphPad Prism software (version 4.0b) (as detailed in figure legends). *p* < 0.05 was considered as statistically significant.

## 3 Results

### 3.1 Imaging of HA-mAPJ in HEK293 cells and the ppERK response to [Pyr^1^]apelin-13

**Fig. 1:**
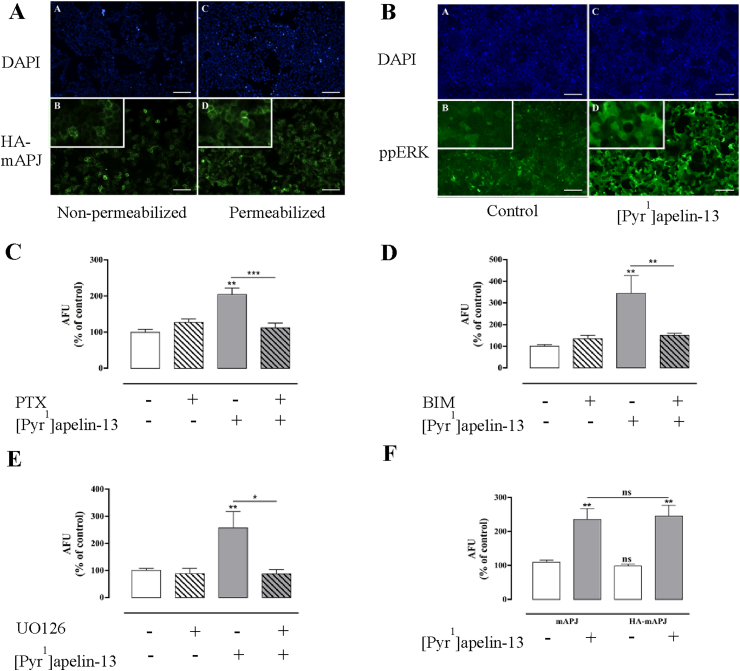
**(A)** Thumbnail images from individual wells stained for HA-mAPJ cell surface and whole cell expression, after stimulation with [Pyr^1^]apelin-13. Representative regions of cell images are shown for DAPI (top panels A and C), and HA-mAPJ (bottom panels B and D) in HA-mAPJ-HEK293 cells with either non-permeabilized (left panels, cell surface) or permeabilized (right panels, whole cell) membranes, higher magnification inset. **(B)** Thumbnail images from individual wells stained for ppERK1/2 expression after stimulation with [Pyr^1^]apelin-13. Representative regions of cell images are shown for DAPI (top panels A and C), and ppERK1/2 (bottom panels B and D) in mAPJ-HEK293 cells stimulated with either vehicle control (left panels) or 100 nM [Pyr^1^]apelin-13 (right panels), higher magnification inset. Scale bars, 100 μm. mAPJ-HEK293 cells were pre-treated with or without **(C)** PTX (200 ng/ml, 16 h), **(D)** BIM (10 μM, 1 h) or **(E)** UO126 (10 μM, 30 min) and stimulated in the presence or absence of [Pyr^1^]apelin-13 (100 nM) for 5 min. **(F)** Tagging of mAPJ with the HA epitope did not interfere with receptor signalling. Non tagged mAPJ-HEK293 cells and HA-tagged mAPJ-HEK293 cells were stimulated with [Pyr^1^]apelin-13 (100 nM) for 5 min and compared with control cells treated with 1× PBS. For **(C**–**F)** cells were fixed, stained, and imaged for determination of whole-cell ppERK1/2 intensity using anti ppERK1/2 antibody. The value determined with no primary antibody present was designated as background and was subtracted from raw data to give arbitrary fluorescence units (AFU) and then normalized to a percentage of vehicle control. Data shown are mean ± SEM, of at least three separate experiments, each with triplicate wells and triplicate fields within wells. *p < 0.05, **p < 0.01, and ***p < 0.001 comparing stimulations to basal conditions, analysed by one-way ANOVA and Dunnett's multiple comparison *post hoc* tests. ns = no statistical significant difference.

To facilitate functional characterization of APJ, a stable HA-mAPJ expressing cell line was generated. In the first experiments receptor expression was confirmed by immunohistochemical detection of the HA tags using automated image acquisition and analysis. As anticipated, essentially all cells expressed HA-mAPJ, that could be detected in permeabilized cells and also when the primary antibody was added to bind the exofacial HA-tag in intact cells (Fig. 1A). We used the pyroglutamyl form of apelin-13, [Pyr^1^]apelin-13, the most potent and abundant form in the brain (De Mota et al., 2004) and cardiovascular system (Maguire et al., 2009), to test for expression of functional receptors and found that 5 min stimulation with 100 nM [Pyr^1^]apelin-13 caused a marked increase in ppERK staining over the cytoplasm and nucleus of mAPJ-HEK293 cells (Fig. 1B). This effect was prevented by pre-treatment with PTX to prevent G_i_ activation; with BIM to prevent PKC activation; or with U0126 to inhibit MEK (Fig. 1C–E). The effects of [Pyr^1^]apelin-13 on ppERK levels in HEK-293 cells expressing non-tagged mAPJ and HA-tagged mAPJ were also compared and were found to be indistinguishable (Fig. 1F).

**Fig. 2:**
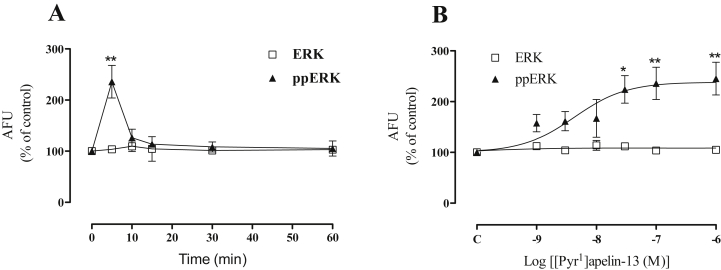
**(A)** [Pyr^1^]apelin-13 stimulated ERK activation in mAPJ-HEK293 at the time points indicated and **(B)** with the indicated concentrations of [Pyr^1^]apelin-13 for 5 min. Cells were fixed, stained, and imaged for determination of whole-cell ppERK1/2 using anti ppERK1/2 antibody, and total ERK1/2 measured with anti-ERK1/2 antibody. The value determined with no primary antibody present was designated as background and was subtracted from raw data to give arbitrary fluorescence units (AFU) and then normalized to a percentage of vehicle control. Data shown are mean ± SEM, of at least three separate experiments, each with triplicate wells and triplicate fields within wells. *p < 0.05 and **p < 0.01 comparing stimulations to basal conditions, analysed by one-way ANOVA and Dunnett's multiple comparison *post hoc* tests.

mAPJ-HEK293 cells were then treated for varied times with [Pyr^1^]apelin-13. The ppERK response was rapid (maximal at 5 min) and transient, reducing to near basal values by 10 min (Fig. 2A). We also varied [Pyr^1^]apelin-13 concentration and this revealed a concentration-dependent effect with an EC_50_ value of ∼3 nM at 5 min (Fig. 2B). No significant variations were seen in total ERK1/2 levels in the mAPJ cell line stimulated with [Pyr^1^]apelin-13 in either time or dose response curves, consequently further experiments were conducted without measurement of total ERK levels.

### 3.2 Homologous and heterologous desensitization of the ppERK response to [Pyr^1^]apelin-13

**Fig. 3:**
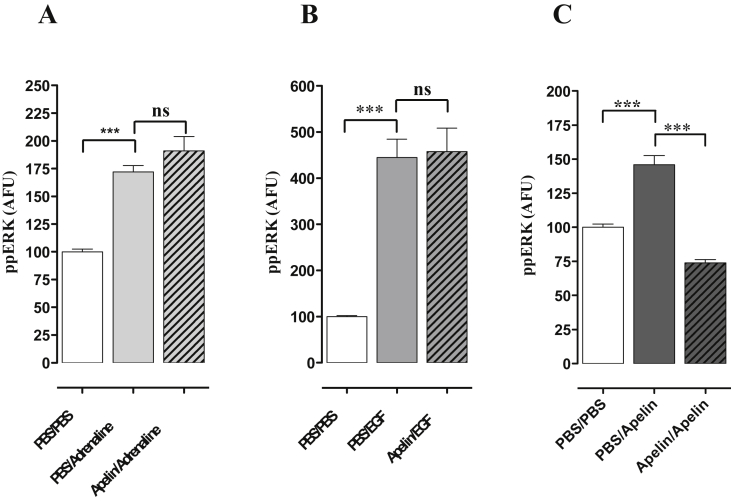
Desensitization of [Pyr^1^]apelin-13-induced ERK1/2 activation in mAPJ-HEK293. mAPJ-HEK293 cells were pre-incubated with PBS or [Pyr^1^]apelin-13 (100 nM, 2 h), washed, and immediately stimulated in the presence of **(A)** adrenaline (1 μM) **(B)** EGF (100 ng/ml) or **(C)** [Pyr^1^]apelin-13 (100 nM) for 5 min. Cells were fixed, stained and imaged for determination of whole cell ppERK1/2 intensity using anti-ppERK1/2 antibody. The value determined with no primary antibody present was designated as background and was subtracted from raw data to give ppERK1/2 intensity in arbitrary fluorescence units (AFU) and then normalized to a percentage of vehicle control. Data shown are mean ± SEM, of at least three separate experiments, each with triplicate wells and triplicate fields within wells. ***p < 0.001, analysed by one-way ANOVA and Dunnett’s multiple comparison *post hoc* tests. ns = no statistical significant difference.

The desensitization of the response to [Pyr^1^]apelin-13 was then investigated using a pretreatment protocol to test for homologous and heterologous desensitization. mAPJ-HEK293 cells were pretreated for 2 h with 0 or 100 nM [Pyr^1^]apelin-13, washed and then immediately stimulated for 5 min with control medium or with medium containing 1 μM adrenaline, 100 ng/ml EGF or 100 nM [Pyr^1^]apelin-13. Adrenaline, EGF and apelin caused robust increases in ppERK in mAPJ-HEK293 control (PBS pre-incubated) cells (Fig. 3A, B and C). As shown (Fig. 3C), 2 h pre-incubation with [Pyr^1^]apelin-13 completely prevented the response to a subsequent 5 min stimulation with [Pyr^1^]apelin-13, but did not measurably alter the responses to adrenaline (Fig. 3A) or EGF (Fig. 3B).

### 3.3 Trafficking of HA-mAPJ

**Fig. 4:**
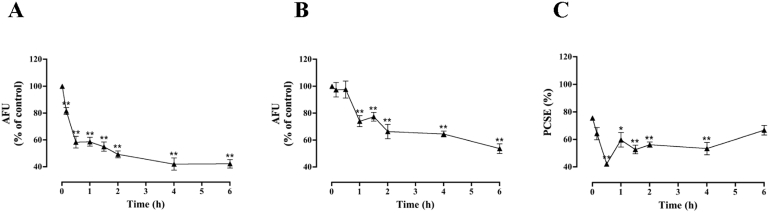
Time course of [Pyr^1^]apelin-13-induced HA-mAPJ localization and expression levels. HA-mAPJ-HEK293 cells were incubated in the presence or absence of [Pyr^1^]apelin-13 (100 nM) for 0–6 h. Cells were fixed, stained and imaged for determination of either **(A)** cell surface or **(B)** whole cell HA-mAPJ intensity using anti-HA antibody. The value determined with no primary antibody present was designated as background and was subtracted from raw data to give HA-mAPJ intensity in arbitrary fluorescence units (AFU) and then normalized to a percentage of vehicle control. Cell surface and whole cell AFU values were used to determine the PCSE **(C)**. Data shown are mean ± SEM, of at least three separate experiments, each with triplicate wells and triplicate fields within wells. * = *p* < 0.05 and ** = *p* < 0.01, comparing stimulations to basal conditions, analysed by one-way ANOVA and Dunnett's multiple comparison *post hoc* tests.

Following the lack of heterologous desensitization described above, that implies that the desensitization of the response to [Pyr^1^]apelin-13 may be due to upstream APJ-specific (rather than down-stream ERK-specific) adaptive mechanisms, we explored possible changes in the amount and compartmentalization of APJ by stimulating HA-mAPJ-HEK293 cells for varied periods (up to 6 h) with 0 or 100 nM [Pyr^1^]apelin-13 before determining cell surface and whole cell HA-mAPJ levels with the intact cell and permeabilized cell staining assays used for Fig. 1. As shown (Fig. 4A), [Pyr^1^]apelin-13 caused a reduction in cell surface HA-mAPJ, which reduced by >50% with a half-time of ∼30 min. It also reduced whole cell HA-mAPJ (Fig. 4B) but the effect was less marked (reduction to ∼60% of control) and slower (no measurable reduction until 1 h). We also used the cell surface and whole cell HA-mAPJ expression measures to calculate the proportional cell surface receptor expression (PCSE; (cell surface expression ÷ whole cell expression) × 100) and found that in control cells ∼76% of HA-mAPJ were at the cell surface and that this reduced to ∼40% after 30 min stimulation with [Pyr^1^]apelin-13 before recovering to near control levels at 6 h (Fig. 4C).

### 3.4 HA-mAPJ internalization and desensitization of APJ-mediated ERK activation

**Fig. 5:**
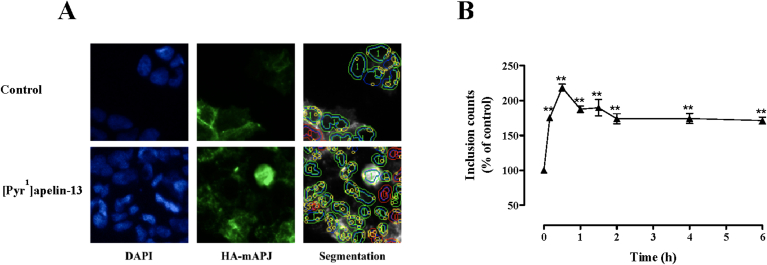
**(A)** Representative regions of cell images shown for DAPI, HA-mAPJ and an illustration of the automated image segmentation used to define perimeters of nuclei (blue) and cells (green or red) and inclusions (yellow) in cells stimulated with control or [Pyr^1^]apelin-13, as indicated. **(B)** Time course of [Pyr^1^]apelin-13–induced HA-mAPJ internalization. HA-mAPJ-HEK293 cells were pre-treated with anti-HA antibody (1:1000; 1 h 37 °C/5% CO_2_), washed, then incubated in the presence or absence of [Pyr^1^]apelin-13 (100 nM) for 0–6 h. Data shown are mean ± SEM, of at least three separate experiments, each with triplicate wells and triplicate fields within wells. **p < 0.01, comparing stimulations to basal conditions, analysed by one-way ANOVA and Dunnett's multiple comparison *post hoc* tests. (For interpretation of the references to colour in this figure legend, the reader is referred to the web version of this article.)

To follow internalization more directly cell surface HA-mAPJ were preloaded with anti-HA antibody in the absence of agonist, and cells were then incubated for varied periods with 0 or 100 nM [Pyr^1^]apelin-13 before determining the number of anti-HA-containing inclusions (presumptive endosomes) by automated image analysis. As shown (Fig. 5A and B), [Pyr^1^]apelin-13 caused a rapid increase with the inclusion count being maximal after 30 min and remaining significantly elevated for 6 h.

**Fig. 6:**
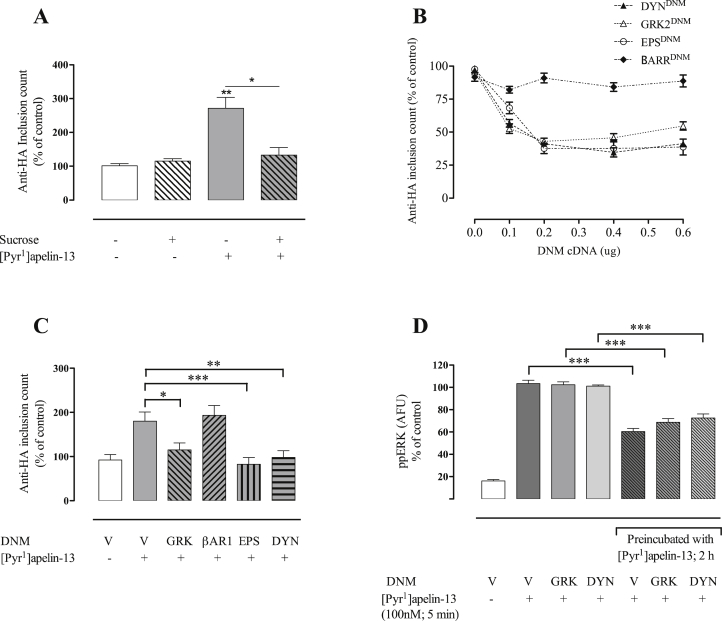
Mechanisms of [Pyr^1^]apelin-13-induced HA-mAPJ internalization and mAPJ desensitization. **(A)** HA-mAPJ-HEK293 cells were pre-treated with anti-HA antibody (1:100; 1 h, 37 °C/5% CO_2_), washed, then incubated in the presence or absence of sucrose (0.4 M) for 45 min, followed by incubation with [Pyr^1^]apelin-13 (100 nM) for 2 h. **(B**, **C)** HA-mAPJ-HEK293 cells were transfected with DNM cDNAs of various CME-related factors or an empty plasmid vector (V). After 48 h, cells were pre-treated with anti-HA antibody (1:100; 1 h, 37 °C/5% CO_2_), washed, incubated in the presence or absence of [Pyr^1^]apelin-13 (100 nM), for 2 h. Cells were fixed, stained and imaged for determination of whole cell inclusion counts, normalized to a percentage of vehicle control. **(B)** shows [Pyr^1^]apelin-13-induced HA-mAPJ internalization after co-transfection with increasing concentrations of GRK^DNM^, EPS^DNM^, DYN^DNM^ and βARR^DNM^. **(C)** shows the effects of GRK^DNM^, EPS^DNM^, DYN^DNM^ and βARR^DNM^ on [Pyr^1^]apelin-13-induced HA-mAPJ internalization. In **(D)** mAPJ-HEK293 cells were transfected with DNM cDNAs of various CME-related factors or an empty plasmid vector (V). After 48 h, cells were washed, incubated in the presence or absence of [Pyr^1^]apelin-13 (100 nM), for 2 h, stimulated with 100 nM [Pyr^1^]apelin-13 for 5 min, fixed, stained and imaged for determination of whole cell ppERK1/2 intensity using anti-ppERK1/2 antibody, expressed as arbitrary fluorescent units (AFU). Data shown are mean ± SEM, of at least three separate experiments, each with triplicate wells and triplicate fields within wells. *p < 0.05, **p < 0.01, ***p < 0.001 analysed by two-way ANOVA and Dunnett's multiple comparison *post hoc* tests.

This assay was also used to explore APJ internalization mechanisms using a 2 h [Pyr^1^]apelin-13 stimulation period. This revealed that pretreatment with hypertonic sucrose to block clathrin-mediated endocytosis completely blocked the [Pyr^1^]apelin-13 effect on inclusion counts (Fig. 6A). The dose-dependent effects of GRK, EPS, DYN and βARR DNMs on HA-mAPJ-HEK293 cells was then assessed. Co-transfection with the individual expression vectors for GRK^DNM^, EPS^DNM^ and DYN^DNM^ inhibited [Pyr^1^]apelin-13-induced HA-mAPJ internalization in a dose-dependent manner, with an optimal concentration of 0.4 µg/well, however the [Pyr^1^]apelin-13-stimulated increase in inclusion count was not blocked by βARR^DNM^ (Fig. 6B). The effects of GRK^DNM^, EPS^DNM^, DYN^DNM^ and βARR^DNM^ on [Pyr^1^]apelin-13-induced HA-mAPJ internalization are shown in Fig. 6C.

We also tested for effects of GRK^DNM^ and DYN^DNM^ cDNAs, both of which prevented HA-mAPJ internalization into inclusions (Fig. 6C), on the desensitization of APJ-mediated ERK activation. Acute (5 min) stimulation of mAPJ-HEK293 cells with [Pyr^1^]apelin-13 caused robust increases in ppERK, that did not alter in cells transfected with GRK^DNM^ or DYN^DNM^ cDNAs (Fig. 6D). Pre-treatment for 2 h with 100 nM [Pyr^1^]apelin-13 caused the expected reduction of subsequent responses to 5 min stimulation with 100 nM [Pyr^1^]apelin-13 in control cells (Fig. 6D, see also Fig. 3) and this reduction was also observed in cells transfected with GRK^DNM^ or DYN^DNM^ cDNAs (Fig. 6D).

### 3.5 Recovery of APJ levels after agonist removal

**Fig. 7:**
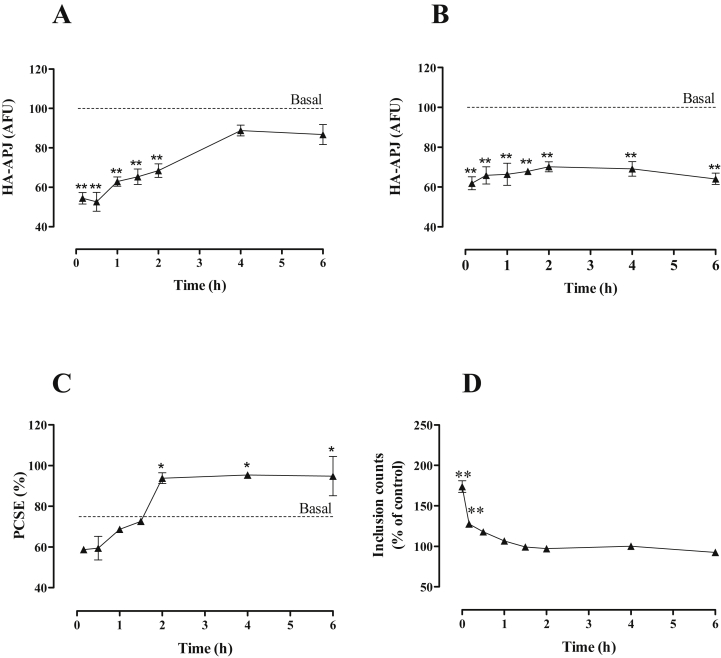
Recovery of APJ levels after agonist removal. HA-mAPJ-HEK293 cells were incubated in the presence or absence of [Pyr^1^]apelin-13 (100 nM) for 2 h, washed, then incubated in fresh medium for 0–6 h. Cells were fixed, stained and imaged for determination of either **(A)** cell surface or **(B)** whole cell HA-mAPJ intensity using anti-HA antibody. The value determined with no primary antibody present was designated as background and was subtracted from raw data to give HA-mAPJ intensity in arbitrary fluorescence units (AFU) and then normalized to a percentage of vehicle control. Cell surface and whole cell AFU values were used to determine the PCSE **(C)**. **(D)** shows recovery from the effect of [Pyr^1^]apelin-13 on HA-mAPJ inclusion count. HA-mAPJ-HEK293 cells were pre-treated with anti-HA antibody (1:100; 1 h, 37 °C/5% CO_2_), washed, incubated in the presence or absence of [Pyr^1^]apelin-13 (100 nM) for 2 h to internalize APJ, washed, then incubated in fresh medium for 0–6 h. Data shown are mean ± SEM, of three separate experiments, each with triplicate wells and triplicate fields within wells. *p < 0.05 and **p < 0.01, comparing stimulations to basal conditions, analysed by one-way ANOVA and Dunnett's multiple comparison *post hoc* tests.

To explore recovery of APJ expression levels following pretreatment with agonist, HA-mAPJ-HEK293 cells were treated for 2 h with 0 or 100 nM [Pyr^1^]apelin-13, washed and allowed to recover for varied periods (0–6 h) before quantification of cell surface HA-mAPJ and whole cell HA-mAPJ levels. As expected, the [Pyr^1^]apelin-13 pre-treatment reduced cell surface and whole cell HA-mAPJ levels by 40–50% (Fig. 7A and B; see also Fig. 4). Cell surface HA-mAPJ levels recovered slowly returning to control levels at 4–6 h after the pre-treatment (Fig. 7A), whereas whole cell HA-mAPJ levels remained low and were essentially unaltered during the 0–6 h recovery period (Fig. 7B). These data were used to calculate PCSE and this was reduced (from an initial ∼76% to ∼60%) by [Pyr^1^]apelin-13 pre-treatment and recovered to almost 100% at 2–6 h after pre-treatment (Fig. 7C). A similar protocol was used to assess recovery from the effect of [Pyr^1^]apelin-13 on HA-mAPJ inclusion count. As expected, pre-treatment for 2 h with [Pyr^1^]apelin-13 increased the number of inclusions by ∼75% and this effect was rapidly reversed so that there was no measurable increase in inclusions after 30 min of recovery (Fig. 7D).

### 3.6 Resensitization of APJ-mediated ERK activation

**Fig. 8:**
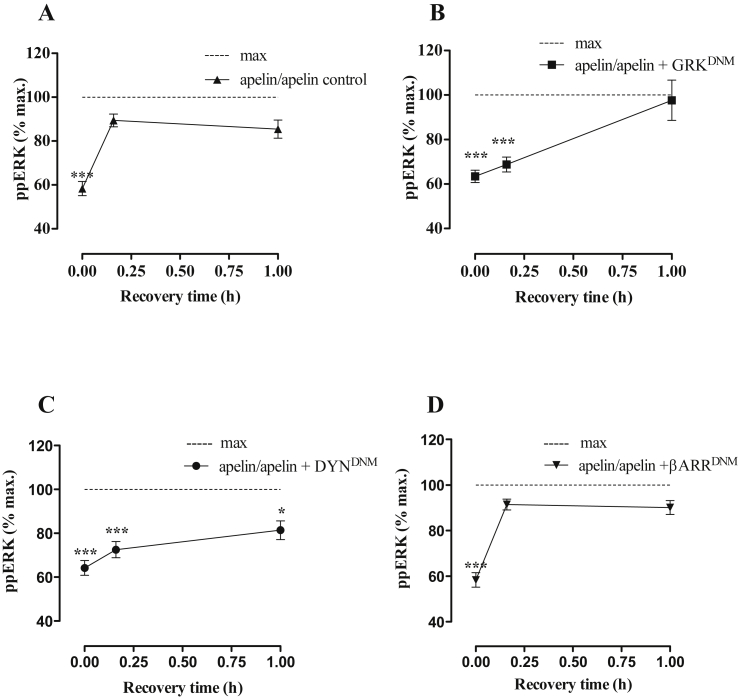
**(A)** mAPJ-HEK293 cells were pre-incubated in the presence or absence of [Pyr^1^]apelin-13 (100 nM, 2 h), washed, incubated in fresh medium for 0–1 h and then stimulated in the presence or absence of [Pyr^1^]apelin-13 (100 nM) for 5 min. Cells were fixed, stained and imaged for determination of whole cell ppERK1/2 intensity using anti-ppERK1/2 antibody. The value determined with no primary antibody present was designated as background and was subtracted from raw data to give ppERK1/2 intensity in arbitrary fluorescence units (AFU) and then normalized to a percentage of vehicle control ([Pyr^1^]apelin-13-induced ERK1/2 signalling in cells initially exposed to vehicle control and designated as “max”). In **(B**–**D)** mAPJ HEK293 cell lines were transfected with GRK^DNM^, DYN^DNM^ or βARR^DNM^ cDNAs respectively before preincubation with or without [Pyr^1^]apelin-13. Data shown are mean ± SEM, of at least three separate experiments, each with triplicate wells and triplicate fields within wells. ***p < 0.001, comparing stimulations to max conditions, analysed by one-way ANOVA and Dunnett's multiple comparison *post hoc* tests.

We then followed recovery from desensitization (of [Pyr^1^]apelin-13-stimulated ERK activation) in control cells and in cells transfected with GRK^DNM^ or DYN^DNM^ cDNAs, both of which prevented HA-mAPJ internalization into inclusions (see Fig. 6), or βARR^DNM^ cDNA. mAPJ-HEK293 cells initially exposed to vehicle control (1× PBS; 2 h) showed significant activation of ERK1/2 after a 5 min exposure to [Pyr^1^]apelin-13. However 2 h pre-treatment of mAPJ-HEK293 cells with 100 nM [Pyr^1^]apelin-13 caused the expected reduction in response to a subsequent 5 min stimulation with 100 nM [Pyr^1^]apelin-13 (Fig. 8A). When cells were allowed to recover for varied periods (0–1 h) before the second stimulus, rapid recovery was observed, with maximal recovery and no measurable desensitization after just 15 min of recovery (Fig. 8A). Recovery was slower in the presence of GRK^DNM^ (Fig. 8B) or DYN^DNM^ cDNAs (Fig. 8C), as for both there was no measurable recovery at 15 min and recovery was near maximal at 1 h. The presence of βARR^DNM^ did not alter the pattern of normal resensitization of [Pyr^1^]apelin-13-induced ERK1/2 activation.

## 4 Discussion

GPCR regulation in response to agonist stimulation is common to nearly all GPCRs and is essential in physiological systems to limit persistent signalling. In this study we have investigated the [Pyr^1^]apelin-13-induced trafficking and desensitization of mAPJ in mAPJ-HEK293 cells using a semi-automated imaging protocol and clearly show that HA-mAPJ internalization is a GRK2-, dynamin- and EPS15-mediated event.

A stable HA-mAPJ expressing cell line was generated and was used to quantify the proportion of APJ at the cell surface and within whole cells using semi-automated acquisition and analysis of digital fluorescence images. While the majority of epitope-tagged mAPJ was localized to the cell surface in these cells, a proportion of tagged APJ was distributed within the cell. This is in contrast to earlier studies that reported enhanced green fluorescent protein (eGFP)-APJ localization, under basal conditions, to be confined to the plasma membrane (El Messari et al., 2004). APJ acts primarily via G_i_ to inhibit adenylyl cyclase but has also been reported to activate other effectors including PKC, PI3K and ERK (Masri et al., 2002, Masri et al., 2004). As positioning of differing tags into the native receptor may have implications for receptor trafficking, we verified that the functional integrity of the receptor in our cell line remained intact and that these HA-mAPJ-HEK293 cells, like their non-tagged counterparts, mediate ERK activation. Significant and similar [Pyr^1^]apelin-13-induced stimulation of ERK1/2 was seen in both HA-tagged and untagged mAPJ transfected HEK293 cells.

Many GPCRs show ligand bias (where different agonists bias signalling toward different effectors) and there is evidence that this may occur for APJ (Masri et al., 2006, Brame et al., 2015). It has been shown recently that the cyclic apelin analogue MM07 displays bias towards stimulation of a beneficial G-protein-dependent pathway, stimulating vasodilation and inotropic actions, over a more damaging G-protein-independent β-arrestin-dependent pathway that results in cardiac hypertrophy (Brame et al., 2015). In this regard, it is also of interest that APJ is most closely related to angiotensin 1 receptors (AT_1_), for which ligand bias has been extensively explored. AT_1A_ receptors are G_q/11_ coupled GPCRs that also activate ERK. They undergo a process of rapid homologous receptor desensitization in which arrestins bind to the activated receptors preventing them from activating their cognate G proteins and targeting them for internalization via CCVs. The arrestins can also act as scaffolds for MAPK cascade components and mediate signalling to ERK. Activation of AT_1A_ receptors can cause an initial phase of G protein-mediated ERK activation followed by a switch to a second phase of arrestin-mediated ERK activation and ligand bias is seen when angiotensin II activates both pathways whereas analogues (such as [Sar(1),Ile(4),Ile(8)]AngII (SII)) engage only the latter (Lefkowitz and Shenoy, 2005, Ahn et al., 2004, Shenoy et al., 2006). We were interested in the possibility that APJ might also mediate such a biphasic response. We established however that when mAPJ-HEK293 cells were treated for varied times with [Pyr^1^]apelin-13, the ppERK response instead was rapid and transient, with an EC_50_ value of ∼3 nM at 5 min, showing no indication of arrestin-mediated activation of ERK1/2.

Having established that the ppERK response to [Pyr^1^]apelin-13 desensitizes rapidly during sustained stimulation in this model, we explored possible mechanisms. Numerous adaptive mechanisms shape ERK responses and these include inhibitory phosphorylation of Ras by ERK (Dumaz and Marais, 2005) or ERK-driven expression of nuclear-inducible dual specificity phosphatases (DUSP) (Caunt et al., 2008). However, neither of these down-stream mechanisms seems likely here as APJ-mediated ERK activation is Ras-independent in CHO cells (Masri et al., 2002) and the desensitization is too fast to be mediated by DUSP neosynthesis (Caunt et al., 2008). We therefore suspected that this rapid desensitization of the response to [Pyr^1^]apelin-13 was due to upstream APJ-specific (rather than down-stream ERK-specific) adaptive mechanisms and explored this by looking at homologous and heterologous desensitization of this response. Desensitization can be homologous or heterologous in nature; homologous desensitization occurs when there is a loss of response solely to an agonist that is acting at one particular GPCR subtype, whereas heterologous desensitization is agonist-non-specific and involves a broad pattern of unresponsiveness at multiple GPCR subtypes. Homologous desensitization is thought to involve adaptive changes at the level of the GPCR itself, whereas heterologous desensitization may also involve altering the efficiency of downstream signalling components. Adrenaline and EGF are known activators of ERK in HEK293 cells, likely acting via the α_1b_ adrenergic (Schonbrunn and Steffen, 2012) and EGF receptors (Kramer et al., 2002) respectively, and as expected, both caused robust increases in ppERK in PBS pre-incubated mAPJ-HEK293 cells, that were not different to the increases seen after pre-incubation with [Pyr^1^]apelin-13. The ppERK response to a subsequent [Pyr^1^]apelin-13 stimulation was however completely abrogated by pre-incubation with [Pyr^1^]apelin-13. These data suggest that the desensitization of [Pyr^1^]apelin-13-induced ERK1/2 phosphorylation was not due to a requirement to reset the intracellular signalling pathway or other post-receptor modifications, but to upstream APJ-specific adaptive changes. These could include receptor internalization, as APJ undergoes agonist-induced internalization via CCVs (Reaux et al., 2001, El Messari et al., 2004), and/or rapid homologous receptor desensitization, as APJ has been shown to cause translocation of β-arrestin to the cell surface in other systems (Lee et al., 2010). We undertook therefore to monitor APJ compartmentalization, and found that incubation of HA-mAPJ-HEK293 cells with [Pyr^1^]apelin-13 decreased both cell surface and whole cell expression of APJ in a time-dependent manner. These data are consistent with agonist-induced receptor internalization followed by degradation of a proportion of the internalized receptors, such that down-regulation follows the reduction in cell surface expression.

As the experimental procedure used could reflect agonist-stimulation of both anterograde and retrograde APJ trafficking, as has been described for the δ-opioid peptide receptor (Zhang et al., 2006b, Zhang et al., 2006a), receptor internalization was more directly monitored by loading cell surface HA-mAPJ with anti-HA antibody before washing and incubation with agonist. We found a rapid increase in [Pyr^1^]apelin-13-induced HA-mAPJ internalization that is consistent with previous confocal microscopy studies showing rapid agonist-induced internalization of eGFP-APJ from the plasma membrane (Lee et al., 2010, El Messari et al., 2004). [Pyr^1^]apelin-13-induced-HA-mAPJ internalization was inhibited in the presence of sucrose, which prevents formation of clathrin-coated pits (Heuser and Anderson, 1989), and by the expression of DNM cDNAs of GRK2, dynamin and EPS15, known effectors of CME, but not by expression of βARR^DNM^. We have used these DNMs successfully to assess GRK2-, β-arrestin1-, dynamin- and EPS-dependent internalization of several GPCRs (e.g. oxytocin receptor (Smith et al., 2006); gonadotropin-releasing hormone receptor (Hislop et al., 2001, Hislop et al., 2005)). The GRK2^DNM^ construct (K220A) reduces agonist-induced GPCR phosphorylation (Mundell et al., 1997); βARR^DNM^ (319–418) competes with wild-type arrestin for clathrin and AP2 binding, and impairs receptor-binding ability (Krupnick et al., 1997); EPS^DNM^ (EΔ95/295) lacks domains recognising EPS15 itself and is required for coated pit formation; and DYN^DNM^ (K44A) inhibits dynamin-mediated scission of CCVs from the plasma membrane (Damke et al., 1994). Together these data suggest that [Pyr^1^]apelin-13 causes internalization of mAPJ via CCVs, and thereby reduces cell surface mAPJ levels. In accord with many other GPCRs, we suggest that GRK2-mediated phosphorylation targets the receptors to CCVs that are internalized from the cell surface in an EPS15- and dynamin-dependent manner. Interestingly, apelin-13-internalized APJ has been reported to dissociate from β-arrestin1 prior to receptor internalization (Lee et al., 2010), and we have shown that the [Pyr^1^]apelin-13 effect on inclusion count was not prevented by transfection with the βARR^DNM^ cDNA. Although not tested, it is likely that both β−arrestin1- and β−arrestin2-mediated endocytosis would be inhibited by transfection of the βARR^DNM^ construct (J.L. Benovic, personal communication). Further work such as using siRNAs specifically against each arrestin will determine whether either is necessary for APJ-mediated internalization. Therefore the means by which these receptors are targeted to CCVs for internalization remains unclear. A similar dynamin-dependent, β-arrestin independent internalization has been reported in the 5-hydroxytryptamine 2A (5-HT_2A_) receptor (Bhatnagar et al., 2001).

Desensitization of APJ has been shown not to occur in C-terminally truncated receptors that lack the majority of the serine and threonine residues that are liable to phosphorylation (Masri et al., 2006). Phosphorylation of GPCRs following ligand activation is the first step in receptor desensitization, occurring rapidly upon exposure to the agonist, and is conducted by second messenger kinases (e.g. protein kinase C (Benovic et al., 1985)) and a family of kinases termed GRKs (Benovic et al., 1986). Transfection of GRK2^DNM^ or DYN^DNM^ cDNA constructs into mAPJ-HEK293 cells pretreated with [Pyr^1^]apelin-13 for 2 h, both of which constructs had prevented HA-mAPJ internalization, did not affect the abrogation of subsequent responses to stimulation with [Pyr^1^]apelin-13. This data suggests that internalization is not a major factor in the desensitization of APJ-mediated ERK activation in these cells, and that desensitization of APJ is not dependent upon phosphorylation of APJ by GRK-2, but likely by kinases other than GRK-2.

Focusing on recovery after agonist removal, pre-incubation of HA-mAPJ-HEK293 cells with [Pyr^1^]apelin-13 and subsequent recovery in agonist-free medium for varied periods revealed that cell surface APJ levels recovered to near control levels at 4–6 h, while APJ levels in the whole cell remained low following up to 6 h of recovery. Similarly, recovery in agonist-free medium rapidly reversed the number of inclusions seen after pre-incubation with [Pyr^1^]apelin-13 for 2 h. These data are generally consistent with agonist-induced internalization and reduction in cell surface HA-mAPJ occurring relatively rapidly during agonist exposure and recovering more slowly after agonist removal. They also reveal two unexpected features. First, the proportion of HA-mAPJ at the cell surface 2 h after removal of the [Pyr^1^]apelin-13 pre-treatment is significantly higher than in control cells prior to [Pyr^1^]apelin-13 pre-treatment (∼98% versus ∼76%), suggesting that all available APJ are specifically compartmentalized at the cell surface upon agonist removal. Second, the rate of loss of inclusions during the recovery period (half-time ∼15 min) was much higher than the rate of recovery of cell surface HA-mAPJ (half-time ∼2 h). This may suggest that inclusions are lost as the anti-HA shifts from early endosomes to a sorting compartment. If so, their loss would be expected to precede recovery of cell surface receptor expression. In following recovery from desensitization we transfected GRK2^DNM^, DYN^DNM^ and βARR^DNM^ cDNAs into mAPJ-HEK293 cells and found delayed resensitization of [Pyr^1^]apelin-13-induced ERK1/2 responses after transfection with GRK2^DNM^ and DYN^DNM^ cDNAs, but not with the βARR^DNM^ cDNA. This indicates that GRK2- and dynamin-dependent receptor internalization may have a role to play in the resensitization of [Pyr^1^]apelin-13-induced ERK phosphorylation, however β−arrestin-dependent internalization is not required for this process. Thus regulation of receptor number may determine responsiveness of mAPJ to repeated [Pyr^1^]apelin-13 stimulation in HEK293 cells.

In summary, these data show that agonist exposure induces internalization and reduction in cell surface HA-mAPJ expression that occurs relatively rapidly during agonist exposure and recovers more slowly after agonist removal. Moreover the ppERK response of mAPJ-HEK293 cells to [Pyr^1^]apelin-13 desensitizes rapidly during sustained stimulation and this desensitization is due to upstream APJ-specific, rather than down-stream ERK-specific, adaptive changes. We show that GRK2-mediated phosphorylation targets mAPJ to CCVs that are internalized from the cell surface in a β−arrestin-independent, EPS15- and dynamin-dependent manner. Our data indicates that receptor internalization is not required for mAPJ desensitization of ppERK responses in the mAPJ-HEK293 cell line. This multifaceted system may be indicative of a complex mechanism in controlling the physiological functions of endogenous apelin and may be important in conditions where there are elevated circulating or tissue levels of APJ. Further study of the signalling and regulation of APJ will help develop ligands for use in potential therapeutic intervention for dysfunctions of physiological homeostasis such as hypertensive disease.

## Conflict of interest

The authors declare that they have no conflict of interest with the contents of this article.

## Author contributions

A-MO’C and GRP conceived and coordinated the research. GRP, ST and A-MO’C designed and performed the research. SJL designed the PCR primers and assisted with generation of the cell lines. GRP, ST, CMcA, SJL and A-MO’C analysed and interpreted the data. All authors contributed to the drafting and revising of the manuscript, and approved the final version of the manuscript.
